# Influence of age on the effect of reduced renal function on outcomes in patients with coronary artery disease

**DOI:** 10.1186/s12889-019-6498-6

**Published:** 2019-02-18

**Authors:** Fei Chen, Zhi-liang Zuo, Fang-yang Huang, Tian-li Xia, Bao-tao Huang, Hua Chai, Qiao Li, Xiao-bo Pu, Yi-yue Gui, Yong Peng, Mao Chen, De-jia Huang

**Affiliations:** Department of Cardiology, West China Hospital, Sichuan University, 37 Guoxue Street, Chengdu, 610041 People’s Republic of China

**Keywords:** Coronary artery disease, Ageing, Renal insufficiency, Prognosis

## Abstract

**Background:**

Ageing is a risk factor for both coronary artery disease (CAD) and reduced renal function (RRF), and it is also associated with poor prognosis in patients with CAD or RRF. However, little is known about whether the impact of RRF on clinical outcomes are different in CAD patients at different age groups. This study aimed to investigate whether ageing influences the effect of RRF on long-term risk of death in patients with CAD.

**Methods:**

A retrospective analysis was conducted using data from a single-center cohort study. Three thousand and two consecutive patients with CAD confirmed by coronary angiography were enrolled. RRF was defined as an estimated glomerular filtration rate (eGFR) of less than 60 ml/min. The primary endpoint in this study was all-cause mortality.

**Results:**

The mean follow-up time was 29.1 ± 12.5 months and death events occurred in 275 cases (all-cause mortality: 9.2%). The correlation analysis revealed a negative correlation between eGFR and age (r = − 0.386, *P* < 0.001). Comparing the younger group (age ≤ 59) with the elderly one (age ≥ 70), the prevalence of RRF increased from 5.9 to 27.5%. Multivariable Cox regression revealed that RRF was independently associated with all-cause mortality in all age groups, and the relative risks in older patients were lower than those in younger ones (age ≤ 59 vs. age 60–69 vs. age ≥ 70: hazard ratio [HR] 2.57, 95% confidence interval [CI] 1.04–6.37 vs. HR 2.00, 95% CI 1.17–3.42 vs. HR 1.46, 95% CI 1.06–2.02). There was a significant trend for HRs for all-cause mortality according to the interaction terms for RRF and age group (RRF*age [≤59] vs. RRF*age [60–69] vs. RRF*age [≥70]: HR 1.00[reference] vs. HR 0.60, 95% CI 0.23–1.54 vs. HR 0.32, 95% CI 0.14–0.75; *P* for trend = 0.010).

**Conclusions:**

RRF may have different impacts on clinical outcomes in CAD patients at different age groups. The association of RRF with the risk of all-cause mortality was attenuated with ageing.

## Background

With the advances in medicine and prolonging of life expectancy, age-related chronic non-infectious diseases have become the major problems affecting human health [1]. The prevalence of coronary artery disease (CAD) increases with ageing, and the morbidity was greatest among persons aged ≥65 years [2]. Chronic kidney disease is also more common in the aged population than in the young [3]. Ageing is a risk factor for the incidence of both disorders. On the other hand, previous studies have demonstrated that ageing is independently associated with outcomes in CAD patients. For patients with acute coronary syndrome (ACS), the risk of in-hospital mortality increased by 70% for every 10-year increment of age [4]. Meanwhile, ageing is also a risk predictor for outcomes in CKD patients. A study conducted in CKD patients showed that the risk of death was higher in the aged than in the young with comparable estimated glomerular filtration rate (eGFR) level [5].

Additionally, CAD patients are also a high-risk population for CKD [6], and reduced renal function (RRF) resulting from CKD is associated with adverse outcomes in these patients. Numerous studies have suggested that even mild renal insufficiency could have a significantly increased risk of death in patients with CAD [7–9]. There is an interaction between CAD and RRF, and ageing is a risk factor for the incidence and poor prognosis of the both. However, little is known about whether the effects of RRF on clinical outcomes are different in CAD patients at different age groups.

This study aimed to investigate whether age influences the effect of RRF on long-term risk of death by analyzing data from 3002 consecutive CAD patients in a single-center cohort study.

## Methods

### Study population

The data source of this investigation was the West China Hospital CAD cohort study. The single-center study prospectively enrolled all patients undergoing coronary angiography with known or highly suspected CAD in West China Hospital, Sichuan University. For this analysis, we included CAD patients consecutively enrolled in the study from July 2008 to January 2012. Patients with CAD were eligible for inclusion in the study if they showed angiographic evidence of ≥50% stenosis in ≥1 coronary vessel. The exclusion criteria included pregnancy, malignancy, severe liver or hematological disorders, and end-stage renal disease (ESRD) with hemodialysis or renal transplant. A total of 3375 CAD patients met these inclusion and exclusion criteria. After further excluding patients with loss of follow-up (*n* = 312) or incomplete follow-up data (*n* = 61), 3002 patients were included in this data analysis. The local institutional review boards approved the study protocol following the Declaration of Helsinki. All subjects have provided written informed consent.

### Baseline characteristics

Baseline demographic and clinical characteristics were obtained from the electronic medical records and reviewed by a trained study coordinator. Blood samples were collected before angiography, and blood biochemistry for kidney function, liver function, blood glucose, serum lipid, cardiac troponin, natriuretic peptide, etc. were analyzed in the Department of Laboratory Medicine of West China hospital. Hypertension was defined in patients with office systolic blood pressure (SBP) ≥ 140 mmHg and/or office diastolic blood pressure (DBP) ≥ 90 mmHg and/or in those receiving antihypertensive drugs. Diabetes mellitus (DM) was defined in patients who had previously undergone lifestyle intervention for diabetes or received antidiabetic medications and/or whose fasting blood glucose level was greater than 7.0 mmol/L or random blood glucose level was greater than 11.1 mmol/L. Patients received medical care according to the clinical practice guidelines; treatment regimens were not affected by their participation in this study.

### Renal function assessment

Serum creatinine (SCr) was measured within the first 24 h after hospital admission prior to coronary angiography using the nonkinetic alkaline picrate (Jaffe) method. Estimate glomerular filtration rate (eGFR) in milliliters per minute per 1.73 m^2^ was calculated using the Modification of Diet in Renal Disease (MDRD) equation [10]. Patients were divided into two stratifications: those without RRF (eGFR ≥60 ml/min, normal or mildly impaired renal function corresponding to strata used to define CKD stages [11]) and those with RRF (eGFR < 60 ml/min, moderately or severely impaired renal function).

### Follow-up and endpoint

We followed all patients from the day of their enrolment until death or January 2013. Follow-up information was collected by contacting patients’ physicians, patients, or their family. All data were corroborated with the hospital medical records. The primary endpoint in this study was all-cause mortality, and the secondary endpoint was cardiac mortality. Death was considered cardiac death if it was caused by acute myocardial infarction, malignant arrhythmias, or heart failure. Sudden unexpected death without another explanation was also considered cardiac death.

### Statistical analysis

Baseline characteristics were compared among patients categorized by age groups and admission eGFR levels. We expressed continuous variables as means ± standard deviation or medians (interquartile range, IQR), as appropriate, and reported categorical variables as counts and percentages. The correlation of eGFR with age was tested by Pearson correlation test. Hazard ratios (HRs) were presented with their 95% confidence intervals (CIs) based on the results of Cox proportional hazards regression models, which we used to investigate the risk effect of RRF on the outcome events in different age groups. The univariate and multivariate models were generated sequentially to determine the successive influence of potential confounders on the relative risk for outcomes by RRF after stratification by age group. We also employed a single Cox regression model including age group (1 reference variable and 2 indicator variables), RRF, the interaction terms for RRF and age group, and other covariates, to further confirm whether the age-related changes in the association of RRF with clinical outcomes existed. Two-sided *P* values of less than 0.05 were considered to indicate statistical significance. We performed all the statistical analyses with the use of SPSS Statistics (version 19.0).

## Results

A total of 3002 patients with CAD were included in this analysis. Their mean age was 64.5 ± 10.6 years. Patients ≤59 or ≥ 70 years of age accounted for 30.5% and 36.7% of the study population, respectively. The mean eGFR was 80.6 ± 40.3 ml/min; 507 (16.9%) patients had eGFR values less than 60 ml/min. Clinical characteristics at presentation differed between the eGFR ≥60 ml/min and eGFR < 60 ml/min groups, with significant variations across age groups (Table 1). In the older groups, there was a higher proportion of female, patients with eGFR < 60 ml/min, and higher incidences of comorbidities such as hypertension, diabetes, and cardiac dysfunction.

**Table 1:** Baseline characteristics of the study population

Characteristics	Age ≤ 59 yrs		Age 60 to 69 yrs		Age ≥ 70 yrs	
No. of patients	eGFR < 60 ml/min	eGFR ≥60 ml/min	eGFR < 60 ml/min	eGFR ≥60 ml/min	eGFR < 60 ml/min	eGFR ≥60 ml/min
	*n* = 54	*n* = 863	*n* = 150	*n* = 833	*n* = 303	*n* = 799
Age, yrs	54.3 ± 4.7	51.4 ± 6.5	65.3 ± 2.7	64.7 ± 2.8	75.6 ± 4.1	74.8 ± 3.8
Gender, female, n (%)	9 (16.7)	84 (9.7)	47 (31.3)	171 (20.5)	106 (35.0)	198 (24.8)
BMI, kg/m^2^	24.9 ± 3.0	24.7 ± 3.0	24.3 ± 3.5	24.2 ± 3.1	24.0 ± 3.1	23.8 ± 3.5
Medical history						
Pre-hypertension, n (%)	37 (71.2)	360 (41.8)	110 (73.8)	471 (56.6)	218 (72.4)	501 (63.0)
Pre-diabetes mellitus, n (%)	11 (21.2)	131 (15.2)	48 (32.0)	188 (22.7)	100 (33.2)	204 (25.6)
At admission						
Systolic blood pressure, mm Hg	130.4 ± 29.4	126.1 ± 21.9	134.4 ± 25.0	130.8 ± 22.0	131.2 ± 23.0	133.7 ± 21.4
Diastolic blood pressure, mm Hg	79.2 ± 20.8	79.0 ± 13.0	77.1 ± 13.7	76.3 ± 11.7	72.2 ± 12.1	74.7 ± 11.8
Heart rate, beats/min	79.0 ± 19.7	73.7 ± 13.2	77.6 ± 16.7	73.4 ± 13.4	75.1 ± 14.9	73.6 ± 13.8
Killip classification ≥ II, n (%)	13 (24.1)	68 (7.9)	21 (16.0)	77 (9.2)	59 (19.5)	107 (13.4)
Laboratory values						
eGFR, ml/min/1.73m^2^	44.8 ± 13.6	93.7 ± 19.3	45.6 ± 13.1	85.5 ± 17.8	46.0 ± 11.7	80.4 ± 15.9
Blood glucose, mmol/L	8.8 ± 5.1	6.7 ± 2.8	8.1 ± 6.0	7.9 ± 3.0	7.7 ± 3.8	7.0 ± 2.8
Total cholesterol, mmol/L	3.9 ± 1.4	4.2 ± 1.3	4.1 ± 1.2	4.0 ± 1.1	4.0 ± 1.0	4.0 ± 1.0
Severity of CAD						
Left main artery, n (%)	2 (3.7)	32 (3.7)	10 (6.7)	44 (5.3)	19 (6.3)	54 (6.8)
Three vessel diseases, n (%)	19 (35.2)	163 (18.9)	57 (38.0)	178 (21.4)	101 (33.3)	245 (30.7)
Diagnosis of ACS, n (%)	34 (63.0)	603 (69.9)	114 (76.0)	617 (74.1)	235 (77.6)	571 (71.5)
Treatment of PCI, n (%)	35 (64.8)	637 (73.8)	89 (59.3)	586 (70.4)	213 (70.3)	549 (68.7)

Data are expressed as means ± SD or counts and percentages, as appropriate

Abbreviations: BMI, body mass index; eGFR, estimated glomerular filtration rate; CAD, coronary artery disease; ACS, acute coronary syndrome; PCI, percutaneous coronary intervention; SD, standard deviation

**Fig. 1:**
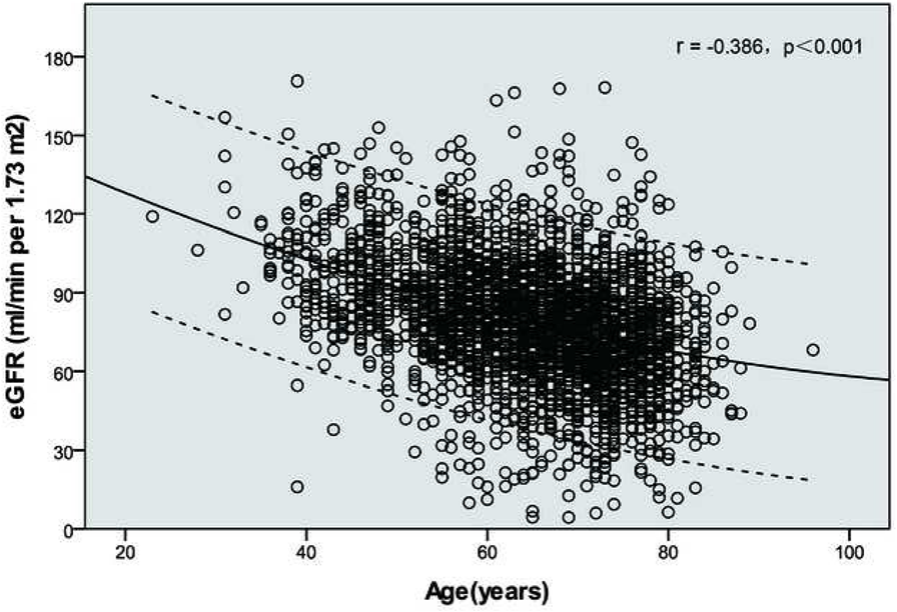
Scatter plot of eGFR and age in years. Abbreviations: eGFR, estimated glomerular filtration rate

**Fig. 2:**
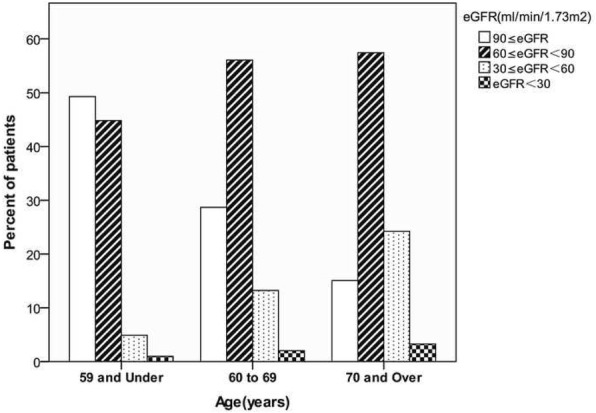
Distribution of eGFR among cohort patients in each age group. Abbreviations: eGFR, estimated glomerular filtration rate

The correlation analysis revealed a negative correlation between the eGFR and age (r = − 0.386, *P* < 0.001) (Fig. 1). Figure 2 shows the distribution of eGFR in patients with CAD among the various age groups. The proportion of patients with an eGFR ≥90 ml/min decreased with ageing. In contrast, the proportions of patients with eGFR ranges of 60 to 90 ml/min, 30 to 60 ml/min and < 30 ml/min gradually increased with ageing. Upon comparing the younger group (age ≤ 59) with the elderly group (age ≥ 70), the eGFR < 60 ml/min prevalence increased from 5.9 to 27.5%.

**Fig. 3:**
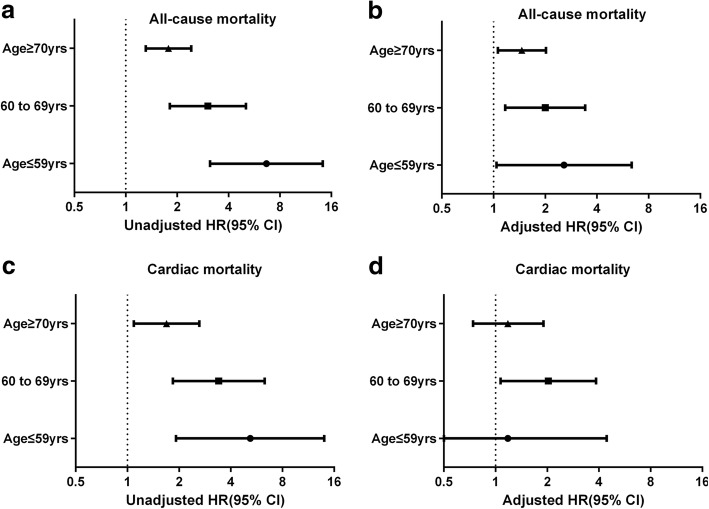
Hazard ratios for mortality by RRF across age groups. Panel **a** and **b**, unadjusted and adjusted hazard ratios for all-cause mortality, respectively; Panel **c** and **d**, unadjusted and adjusted hazard ratios for cardiac mortality, respectively. RRF means eGFR < 60 ml/min per 1.73 m^2^. Adjusted models include covariates as follows: RRF, age, gender, pre-hypertension, pre-diabetes mellitus, systolic blood pressure, Killip class, severity of CAD, diagnosis of ACS, and treatment of PCI. Abbreviations: eGFR, estimated glomerular filtration rate; CAD, coronary artery disease; ACS, acute coronary syndrome; PCI, percutaneous coronary intervention

Of the 3002 patients studied, the mean follow-up period was 29.1 ± 12.5 months; 275 cases of death (all-cause mortality rate: 9.2%) were reported, with 152 cases of death due to cardiovascular events (cardiac mortality rate: 5.1%). The unadjusted analysis showed that, regardless of age groups, RRF (eGFR < 60 ml/min) was associated with higher all-cause mortality in the study population. However, the association was weaker among the older patients than among the younger ones (age ≤ 59 vs. age ≥ 70: hazard ratio [HR] 6.67, 95% confidence interval [CI] 3.12–14.25 vs. HR 1.78, 95% CI 1.81–5.05). After multivariable adjustment, RRF remained independently associated with all-cause mortality in all age groups, and the relative risks in the older patients were lower than those in the younger ones (age ≤ 59 vs. age 60–69 vs. age ≥ 70: HR 2.57, 95% CI 1.04–6.37 vs. HR 2.00, 95% CI 1.17–3.42 vs. HR 1.46, 95%CI 1.06–2.02) (Table 2 and Fig. 3). The unadjusted Cox regression analysis for cardiac mortality was similar to that for all-cause mortality. However, RRF was not correlated with cardiac mortality after multivariate adjustment in the age ≤ 59 and the age ≥ 70 groups, possibly because of the lack of statistical power due to a small number of patients with cardiac death (Table 2 and Fig. 3).

**Table 2:** Risk for outcomes by RRF after stratification by age group

Age group	Age ≤ 59 yrs	Age 60 to 69 yrs	Age ≥ 70 yrs
Outcomes	HR (95% CI)	HR (95% CI)	HR (95% CI)
All-cause mortality			
Unadjusted	6.67 (3.12–14.25)	3.03 (1.81–5.05)	1.78 (1.31–2.42)
Adjusted			
Model 1^a^	5.03 (2.32–10.89)	2.79 (1.67–4.67)	1.68 (1.23–2.28)
Model 2^b^	4.83 (2.22–10.51)	2.73 (1.63–4.58)	1.76 (1.29–2.39)
Model 3^c^	2.57 (1.04–6.37)	2.00 (1.17–3.42)	1.46 (1.06–2.02)
Cardiac mortality			
Unadjusted	5.19 (1.92–14.00)	3.40 (1.84–6.30)	1.69 (1.09–2.62)
Adjusted			
Model 1^a^	4.08 (1.49–11.18)	3.13 (1.69–5.80)	1.56 (1.00–2.42)
Model 2^b^	3.85 (1.40–10.64)	3.09 (1.66–5.75)	1.65 (1.06–2.57)
Model 3^c^	1.18 (0.31–4.44)	2.03 (1.07–3.85)	1.18 (0.74–1.90)

RRF means eGFR<60 ml/min/1.73 m2

^a^ Model 1 adjusted for age; ^b^ Model 2 adjusted for factors of Model 1 and gender; ^c^ Model 3 adjusted for factors of Model 2 and other factors: pre-hypertension, pre-diabetes mellitus, systolic blood pressure, Killip class, severity of CAD, diagnosis of ACS, and treatment of PCI

Abbreviations: *RRF* reduced renal function; *eGFR* estimated glomerular filtration rate, *HR* hazard ratio, *CI* confidence interval, *CAD* coronary artery disease, *ACS* acute coronary syndrome, *PCI* percutaneous coronary intervention

The results of Cox regression analysis including interaction terms for age group and RRF showed that there was a significant trend for HRs for all-cause mortality according to the interaction terms even after multivariable adjustment (RRF*age [≤59] vs. RRF*age [60–69] vs. RRF*age [≥70]: HR 1.00[reference] vs. HR 0.60, 95% CI 0.23–1.54 vs. HR 0.32, 95% CI 0.14–0.75; *P* for trend = 0.010) (Table 3), which further confirmed the existence of age-related attenuation in the association between RRF and all-cause mortality. However, the results of HRs for cardiac mortality according to the interaction terms were not statistically significant after multivariable adjustment.

**Table 3:** Hazard ratios for outcomes according to the interaction terms for age group and RRF

Outcomes	All-cause mortality	Cardiac mortality
Models	HR (95% CI)	HR (95% CI)
Model 1		
RRF*age (≤59)	1.00(reference)	1.00(reference)
RRF*age (60–69)	0.46 (0.19–1.15)	0.50 (0.67–2.14)
RRF*age (≥70)	0.27 (0.12–0.61)	0.33 (0.11–0.97)
P for trend	0.003	0.048
Model 2		
RRF*age (≤59)	1.00(reference)	1.00(reference)
RRF*age (60–69)	0.60 (0.23–1.54)	1.18 (0.34–4.16)
RRF*age (≥70)	0.32 (0.14–0.75)	0.48 (0.15–1.55)
P for trend	0.010	0.053

Model 1 includes age group, RRF (eGFR < 60 ml/min/1.73 m2), and the interaction terms for them (RRF*age group)

Model 2 includes age group, RRF (< 60 ml/min/1.73 m2), the interaction terms for them (RRF*age group), and further adjusted for gender, pre-hypertension, pre-diabetes mellitus, systolic blood pressure, Killip class, severity of CAD, diagnosis of ACS, treatment of PCI

Abbreviations: *RRF* reduced renal function; *eGFR* estimated glomerular filtration rate, *HR* hazard ratio, *CI* confidence interval, *CAD* coronary artery disease, *ACS* acute coronary syndrome, *PCI* percutaneous coronary intervention

## Discussion

This study showed that 1) RRF was more prevalent in the aged CAD patients than in the young ones; 2) RRF might have different impacts on clinical outcomes in CAD patients at different age groups, and the association of RRF with the relative risk of all-cause mortality was attenuated with ageing.

According to the US National Health and Nutrition Evaluation Survey (NHANES), the prevalence of CAD was 46.8% among individuals over 70 years and older, while the prevalence was only 6.71% among individuals between 40 and 59 years old [12]. Observational studies have revealed that, with ageing, nearly two-thirds of “healthy” seniors will experience impaired renal function (measured by SCr clearance) [13]. The present study found that eGFR was inversely correlated with age in CAD patients, which was consistent with the result of the survey conducted in the general population [13]. Currently, the reason for such a correlation remains unclear. Some researchers considered this finding as a consequence that is affected by multiple factors, such as blood vessel stiffness and senescence. CAD is often associated with multiple risk factors for arteriosclerosis; thus, patients with CAD are often complicated by RRF [14]. With ageing, renal vascular and glomerular sclerosis and renal tubular atrophy may be present; renal function may decrease from year to year and will gradually progress into mild renal insufficiency without evident clinical symptoms and signs; moreover, renal insufficiency will be further exacerbated with ageing [11, 15].

Renal insufficiency is considered an important risk factor for cardiovascular events and death in CAD patients [14, 16]. Aged patients may present with a variety of risk factors for adverse outcomes of cardiovascular diseases, such as hypertension, high cholesterol, and diabetes. When CAD is complicated by renal insufficiency in the aged patients, the effects of these risk factors become superimposed. Thus, renal insufficiency may have different effects on the prognoses of patients with CAD in different age groups. The present study showed that the relative risk for death of moderate to severe renal insufficiency was higher in the younger CAD patients than in the older ones, which was consistent with the study conducted in the general population [17]. This association was also found in patients with hypertension, subclinical hypothyroidism, and other chronic diseases [18, 19]. Age plays a vital role as an effect modifier in terms of the relation between conditions and prognosis. The relation between risk factor and mortality may vary across different age groups.

Currently, the mechanism underlying the role of age-related differences in the prognostic significance of renal insufficiency remains unclear. Several factors may be involved. First, aged patients may present with multiple risk factors of death, which may attenuate the relative risk of renal insufficiency; conversely, younger patients are relatively healthy, and thus, a single risk factor (such as RRF) may weigh more in the risk of death [17, 20]. Second, as mentioned above, some “healthy” aged patients may have renal insufficiency [13]. It is controversial to consider this phenomenon as a pathological process or a “normal” ageing process. Furthermore, its impact on prognosis is inconclusive. Third, the Modification of Diet in Renal Disease (MDRD) equation has not been well validated in the aged patients [21], and the low accuracy of the SCr-based equation has been reported [15]. Therefore, it should be made clear that there may be age-related difference in the accuracy of the equation for estimating the actual glomerular filtration rate when using the MDRD equation in the aged patients.

It is a common clinical issue that aged CAD patients are complicated by renal insufficiency. However, current studies cannot provide enough and reliable evidence to guide the medical care for these special populations in clinical practice due to the lack of high-quality data [22, 23]. The guidelines for CKD described the recommended regimens based on the eGFR classification, and the same criteria were applied to both aged and young patients [11]. However, existing studies have suggested that there may be differences in diagnostic and prognostic aspects of CKD in patients at different age groups, and the management strategy considering age stratification may be more reasonable, though available evidence is not currently enough [15, 20]. Our study explored this issue in a real-world CAD cohort. The results suggested that age stratification and the interaction for age and RRF may be important in the management of patients with CAD and renal insufficiency. This study may provide some preliminary evidence and clues for future high-quality researches.

The limitations of this study were as follows. First, the present study was a single-center observational study. Although we performed multivariable adjustments, it is difficult to avoid selection bias and exclude confounding factors entirely. Second, the sample size of this study was relatively small, and it was impossible to run further stratified analyses due to the small number of the over-aged patients and patients with severely RRF (eGFR < 30 ml/min). Third, the SCr level was measured only once on admission; therefore, measurement errors may exist. However, in observational studies, it is impractical to perform a repeated test for SCr levels in the absence of clinical needs, which is an inherent limitation of the real-world researches. At last, as previously described, the accuracy of the eGFR may be insufficient in aged patients by using the SCr level and the MDRD equation.

## Conclusions

RRF may have different impacts on clinical outcomes in CAD patients at different age groups. The association of RRF with the risk of all-cause mortality was attenuated with ageing. This result demonstrated that age stratification is important in the treatment for patients with CAD and RRF. Regarding aged patients with CAD and RRF, the comprehensive assessment for the impacts of other risk factors on clinical outcomes should be considered, along with the attention paid to RRF.

## Abbreviations

ACS: Acute coronary syndrome
BMI: Body mass index
CAD: Coronary artery disease
CI: Confidence interval
CKD: Chronic kidney disease
DBP: Diastolic blood pressure
DM: Diabetes mellitus
eGFR: Estimated glomerular filtration rate
ESRD: End-stage renal disease
HR: Hazard ratio
MDRD: Modification of Diet in Renal Disease
OR: Odds ratio
PCI: Percutaneous coronary intervention
RRF: Reduced renal function
SBP: Systolic blood pressure
SCr: Serum creatinine
SD: Standard deviation
